# On the Susceptibility of Reinforced Concrete Beam and Rigid-Frame Bridges Subjected to Spatially Varying Mining-Induced Seismic Excitation

**DOI:** 10.3390/ma17020512

**Published:** 2024-01-21

**Authors:** Paweł Boroń, Izabela Drygała, Joanna Maria Dulińska, Szymon Burdak

**Affiliations:** 1 Faculty of Civil Engineering, Cracow University of Technology, 31-155 Cracow, Poland; pboron@pk.edu.pl (P.B.); jdulinsk@pk.edu.pl (J.M.D.); 2 TechnipFMC Kraków, 31-864 Cracow, Poland; burdak.szymon@gmail.com

**Keywords:** reinforced concrete bridges, bridge dynamics, beam bridge, rigid-frame bridge, spatially varying earthquake ground motion, mining-induced seismicity, quasi-static response

## Abstract

This paper aims to identify the optimal reinforced concrete bridge construction for regions at risk of mining-induced seismic shocks. This study compares the performances of two common bridge types made of the same structural tissue, i.e., a reinforced concrete beam bridge and rigid-frame bridge under real mining-induced tremors using uniform and spatially varying ground motion models. This study investigates the dynamic responses of the bridges depending on wave velocity and assesses their susceptibility to mining-triggered tremors based on the contribution of quasi-static and dynamic effects in the global dynamic responses of the bridges. This study revealed significant changes in dynamic response under spatially varying ground excitation for both bridge types. It was observed that rigid-frame bridges show higher susceptibility to quasi-static effects due to their stiffness, whereas beam bridges are more susceptible to dynamic stresses. This study recommends that in regions with mining tremors, the choice between bridge types should consider the possibility of limiting individual components of stress. A solution may involve the reduction in quasi-static components through structural reinforcement or decreasing dynamic components by using vibration absorbers. It was found that beam bridges are more cost-effective and practical in mining-affected areas, especially when founded on weak grounds.

## 1. Introduction

Mining activity areas are typically characterized by extensive industrialization and well-established road infrastructure, including numerous multi-span bridges and viaducts. In contemporary mining regions, bridges are mainly constructed of reinforced concrete and incorporate two principal structural systems: the beam and the rigid-frame structure. Design guidelines for these reinforced concrete bridges are collected in the “Catalog of typical structures of road bridges and culverts” [1]. The solutions outlined in the catalog enable the standardization of bridges in terms of construction, reinforcement percentage, and concrete class. This standardized approach ensures the robustness and adaptability of the reinforced concrete bridge to meet the requirements of European Standards and, at the same time, ensure the reliability required for this type of engineering facility.

Considering the dynamic loads resulting from mining-induced seismic tremors is important in designing and maintaining of bridge structures situated in regions with the mining-based exploitation of natural resources. Mining-triggered tremors exhibit distinctive features that differentiate them from natural seismic shocks [2,3,4,5,6,7]. These differences are evident in various aspects, including the dominant frequency range, shock duration, and likelihood of recurrence. Nevertheless, the methods used to calculate the dynamic performances of structures under natural earthquakes and mining tremors are similar.

In the case of long structures like bridges, it is crucial to account for the spatial variability in earthquake ground motion in terms of both amplitude and frequency [8,9,10]. Eurocode 8 [11] recommends considering this effect for long bridge structures under seismic events. The spatially varying ground motion phenomenon is well recognized in regions with natural seismicity [12,13,14], but, to the best of the authors’ knowledge, it is rarely referred to in the context of mining-induced tremors [15,16,17]. Also, most studies of bridges and other multiple-supports structures focus on their responses to spatiotemporal variation in ground motion resulting from natural earthquakes [18,19,20,21,22,23,24,25,26,27,28,29].

Studies by Parvanehro et al., Papadopoulos et al., Kumar, and Bakhshizadeh et al. [18,19,20,21] illustrate various approaches used to estimate the responses of bridges to spatially varying kinematic excitation. The authors explored the main phenomena contributing to the non-uniformity of ground motion, such as the wave passage effect, coherency loss, and the site effect. By comparing the results obtained from analyses using non-uniform excitation to those obtained through the classical approach with uniform excitation, it was possible to determine the impacts of non-uniformity effects on the structural dynamic response. Through an analysis conducted on a wide range of structures varying in length, number of supports, stiffness of piers, and type of soil beneath the supports, the authors assessed the susceptibility of bridge structures to non-uniform ground motion, considering various design solutions and external factors. The results obtained for the analyzed bridges made of the same structural tissue revealed that the application of a non-uniform kinematic excitation model leads to significantly different outcomes compared to the classical uniform model. The studies showed that, for some structural elements, the application of a non-uniform excitation model resulted in lower stresses or displacements than in the case of uniform excitation.

Lupoi et al. [22] analyzed a 200-meter-long four-span bridge with varied pier heights and deck stiffnesses, affected by asynchronous excitation in the longitudinal direction only. The study showed that asynchronous motion can increase the probability of bridge failure. Subsequently, Tzanetos et al. [23] investigated a four-span bridge with a total length of 184 m exposed to non-uniform ground motion. The inelastic analysis showed a significant increase in the dynamic response, especially in the transverse direction. The authors identified the suppression of the fundamental mode and dominance of higher-mode response as distinctive features of asynchronous excitation. Monti et al. [24] conducted research on the inelastic response of a six-span reinforced concrete bridge. The authors demonstrated that the bridge responded almost entirely pseudostatically to non-uniform excitation. Additionally, Burdette et al. [25], and Sextos & Kappos [26] found that asynchronous excitation triggers higher, mainly anti-symmetric modes of vibration.

In turn, research on shaking tables on scaled models [27,28] confirmed that the application of non-uniform excitation can lead to a significant increase in the dynamic responses of bridge structures. It is also worth referring to an economic analysis that considers the costs of repairing selected bridges that would arise due to neglecting the effects of non-uniformity during the design of structures [29]. The authors demonstrated that the repair costs for a bridge subjected to non-uniform seismic excitation can be nearly twice as high as in the case of a response to uniform shock. It is evident, therefore, that the effects of non-uniform excitation cannot be neglected.

The lack of literature concerning the assessment of the dynamic responses of bridges with different structural systems subjected to spatially varying seismic excitation of mining origin inspired the authors of this paper to address this topic.

The primary objective of the article was to identify the optimal construction of the reinforced concrete bridge, i.e., one characterized by lower susceptibility to spatially varying ground excitations, for regions experiencing mining-induced seismic shocks. Two typical bridge constructions were taken into consideration: a beam and a rigid-frame bridge. Both bridge structures considered in this study are based on the “Catalog of typical structures of road bridges and culverts” [1]. The Catalog is presented and recommended for use by bridge engineers on the government website https://www.gov.pl/web/infrastruktura/katalog-typowych-konstrukcji-drogowych-obiektow-mostowych-i-przepustow2 (accessed on 15 January 2024).

The novelty of the paper is found in the following areas: (1) the comparative analyses of the chosen bridges’ performances under a real mining-induced tremor, using two different excitation models, i.e., the uniform and spatially varying ground motion models; (2) the investigation of the dependence of the overall dynamic responses of the bridges, as well as quasi-static and dynamic components, on the wave velocity; (3) the assessment of the susceptibility of two distinctly designed bridges to mining-triggered tremors, based on the contribution of quasi-static and dynamic effects to the global dynamic response.

## 2. Materials and Methods

### 2.1. Characteristics of Mining-Induced Tremors and the Shock Used for Dynamic Analysis

Mining-induced tremors reveal fundamental characteristics that distinguish them from natural seismic shocks. These shocks typically present higher values in the dominant frequency band (2–7 Hz) compared to the frequency contents of natural seismic events (0.5–2 Hz) [30]. They are also notably shorter than seismic events [31]. While earthquakes may have an intense phase lasting in minutes, mining tremors confine this period to a range of 0.5 to 5 s.

In the evaluation of seismic influences, the primary focus is directed towards the horizontal component of vibrations aligned with the direction of wave propagation, resulting from the horizontal component of the Rayleigh wave. Calculations of the response of objects to natural earthquakes often only focus on this component, neglecting others. In the context of mining-induced seismicity, the source is in close proximity to the receiver, allowing all types of body waves (P, SV, and SH), along with surface waves, to arrive almost simultaneously. As a result, vertical amplitudes may even exceed those of horizontal vibrations, indicating that all components must be taken into consideration [32]

Mining tremors are limited to much smaller areas compared to earthquakes [33]. The acceleration time traces exhibit considerable irregularity, with both acceleration and frequency undergoing fluctuations over time. Their amplitudes frequently display an impulse-like nature characterized by rapidly decaying vibrations. The reductions in the vibration amplitudes with increasing distance from the source occur much more rapidly compared to the attenuation observed in the case of natural earthquakes. Therefore, the spatial variation in kinematic excitation may significantly impact the dynamic performances of long structures. A concise summary of the differences between the natural and mining-induced seismic shocks is given in Table 1.

In summary, when analyzing the dynamic responses of extensive structures like bridges to tremors induced by mining activities, it is crucial to (1) account for the three components of a shock and (2) incorporate a model of kinematic excitation that captures its spatial variation.

One of the main regions in Poland exhibiting a significant intensity of mining-induced tremors is the Upper Silesian Coal Basin (USCB). Annually, more than 500 tremors occur in the USCB. The typical energy associated with these mining tremors falls within the range of 10^5^ to 10^7^ J, although events with energy levels up to 10^10^ J have been documented [5,6,34,35]. In the USCB region, the recorded maximum amplitudes of these tremors can reach values of up to 1.7 m/s^2^, and their predominant frequencies typically range from 3 to 8 Hz [36,37].

**Figure 1:**
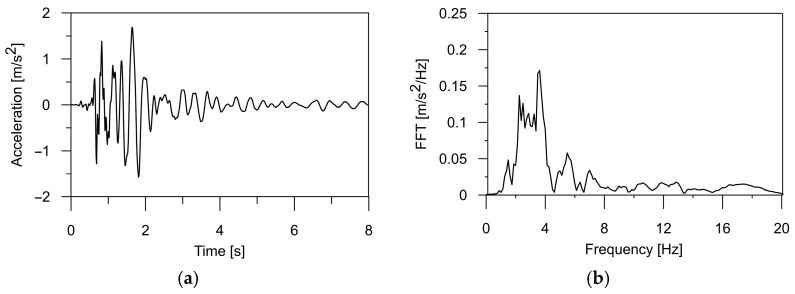
(**a**) The time history of the ground acceleration registered in the USCB region in horizontal direction WE and (**b**) its frequency spectrum.

**Figure 2:**
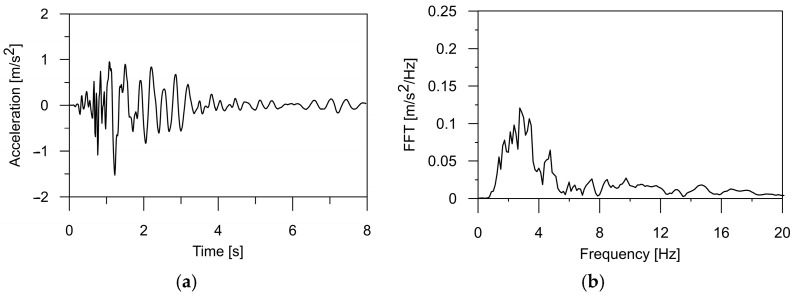
(**a**) The time history of the ground acceleration registered in the USCB region in horizontal direction NS and (**b**) its frequency spectrum.

**Figure 3:**
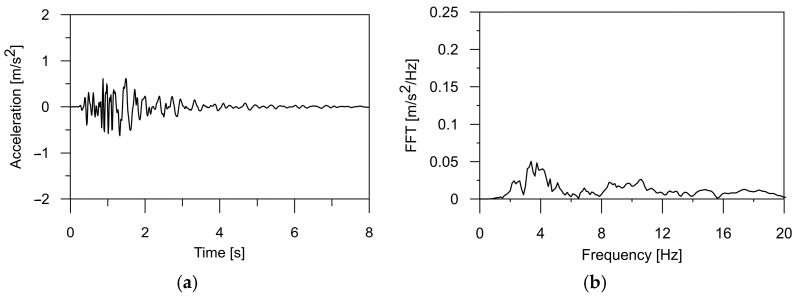
(**a**) The time history of the ground acceleration registered in the USCB region in vertical direction and (**b**) its frequency spectrum.

In this paper, the authors applied a typical mining tremor recorded in the USCB as the kinematic excitation for the structure [32]. The shock acceleration was registered in three directions (two horizontal and one vertical), and the corresponding frequency spectrum is illustrated in Figure 1, Figure 2 and Figure 3. The intense phase of the shock lasted about 3.5 s, and the energy of the shock was $1\cdot 10^{7}$ J. The maximum value of the horizontal acceleration reached 0.35 m/s^2^ in the WE and 0.28 m/s^2^ in the NS directions, respectively. In the vertical direction, accelerations of 0.12 m/s^2^ were observed. Spectral analysis of the shock revealed dominant frequencies of 3.56 Hz in the WE direction, 2.8 Hz in the NS direction, and 3.4 Hz for the vertical component of the shock. For the purposes of this analysis, the mining tremor accelerations were scaled up to the maximum value of PGA = 5·0.35 m/s^2^, resulting in 1.75 m/s^2^. This value corresponded to the most intense tremors experienced in the region.

### 2.2. Theoretical Framework for the Dynamic Response of Multiple-Support Structures to Spatially Varying Ground Motion

For the multi-degree-of-freedom system subjected to multiple-support excitation, the equation of motion could be written as follows [38,39]:

(1) $$\left[\begin{array}{cc} M_{ss} & M_{sg}\\ M_{gs} & M_{gg} \end{array}\right]\cdot \left(\begin{array}{c} {\ddot{x}}_{s}\\ {\ddot{x}}_{g} \end{array}\right)+\left[\begin{array}{cc} C_{ss} & C_{sg}\\ C_{gs} & C_{gg} \end{array}\right]\cdot \left(\begin{array}{c} {\dot{x}}_{s}\\ {\dot{x}}_{g} \end{array}\right)+\left[\begin{array}{cc} K_{ss} & K_{sg}\\ K_{gs} & K_{gg} \end{array}\right]\cdot \left(\begin{array}{c} x_{s}\\ x_{g} \end{array}\right)=\left(\begin{array}{c} 0\\ P_{g} \end{array}\right)$$

where

**M**_ss_, **C**_ss_, and **K**_ss_—mass, damping, and stiffness matrix corresponding to non-support DOFs;
**M**_gg_, **C**_gg_, and **K**_gg_—mass, damping, and stiffness matrix corresponding to support DOFs;
**M**_sg_, **C**_sg_, and **K**_sg_—mass, damping, and stiffness coupling matrix;
${\ddot{x}}_{s}$,$\;{\dot{x}}_{s}$, and $x_{s}$—accelerations, velocities, and displacements vector for each of the DOFs of the structure;
${\ddot{x}}_{g}$, ${\dot{x}}_{g}$, and $x_{g}$—accelerations, velocities, and displacements vector for each of the DOFs of the ground;
$P_{g}$—forces generated at the supports.

In the case of multiple-support excitation, the vector of the total displacements of a structure x could be expressed as the sum of dynamic components, with $x_{s}^{d}$ representing the structure’s displacements relative to the supports, and quasi-static displacements $x_{s}^{q}$ were induced by the quasi-static motion of the supports $x_{g}^{q}$. To simplify the analysis, the dynamic component of soil displacements is commonly neglected [38,39,40], and then the vector of the total displacements was given as:

(2) $$x=\left(\begin{array}{c} x_{s}^{d}\\ 0 \end{array}\right)+\left(\begin{array}{c} x_{s}^{q}\\ x_{g}^{q} \end{array}\right)$$

The quasi-static displacement of the structure was determined under the assumption that the ground displacements were applied infinitely slowly. In this case, the mass and damping matrices disappeared, and Equation (1) was simplified as follows:

(3) $$x_{s}^{q}=-K_{ss}^{-1}\cdot K_{sg}\cdot x_{g}.$$

Substituting Equations (2) and (3) into Equation (1) led to the following equation:

(4) $$M_{ss}\cdot {\ddot{x}}_{s}^{d}+C_{ss}\cdot {\dot{x}}_{s}^{d}+K_{ss}\cdot x_{s}^{d}=-(M_{ss}\cdot R+M_{sg})\cdot {\ddot{x}}_{g}$$

This was obtained after reducing the comparatively negligible term $(C_{ss}\cdot R-C_{sg})\cdot {\dot{x}}_{g}$, as is recommended by the Eurocode 8 [41].

To solve Equation (4), the time history of ground accelerations ${\ddot{x}}_{g}$ was applied to simulate the motion of the supports. In the case of multiple-support structures, it was crucial to recognize that the ground excitation could differ at various support locations. Therefore, excitation models describing ground movement were used, considering effects such as wave passage, coherency loss, and site effects.

### 2.3. Theorethical Basis of the Large Mass Method

The Large Mass Method (LMM) [42] was one of the approaches used to determine the dynamic response of a structure to the multiple-support excitation. The fundamental assumption of the LMM is to transform the kinematic excitation of the system with mass **M_ss_**, represented by the movement of the supports acceleration ${\ddot{x}}_{g}$ into the inertia forces **P_0_**. This conversion is accomplished by incorporating a significant mass **M_0_** to the degrees of freedom supporting the structure and the equivalent kinematic excitation ${\ddot{x}}_{0}$. Therefore, Equation (1) took the following form:

(5) $$\left[\begin{array}{cc} M_{ss} & M_{sg}\\ M_{gs} & M_{gg}+M_{0} \end{array}\right]\cdot \left(\begin{array}{c} {\ddot{x}}_{s}\\ {\ddot{x}}_{g} \end{array}\right)+\left[\begin{array}{cc} C_{ss} & C_{sg}\\ C_{gs} & C_{gg} \end{array}\right]\cdot \left(\begin{array}{c} {\dot{x}}_{s}\\ {\dot{x}}_{g} \end{array}\right)+\left[\begin{array}{cc} K_{ss} & K_{sg}\\ K_{gs} & K_{gg} \end{array}\right]\cdot \left(\begin{array}{c} x_{s}\\ x_{g} \end{array}\right)=\left(\begin{array}{c} 0\\ P_{0} \end{array}\right)$$

where

**M**_0_—large mass added to supported DOFs;
$P_{0}=M_{0}\cdot {\ddot{x}}_{0}$—inertia forces;
${\ddot{x}}_{0}$—vector of large mass acceleration.

When **M_0_** >> **M_ss_**, the displacements of large mass $x_{0}$ were very close to the displacements $x_{g}$ [43]. It was estimated that the applied mass should be approximately 10^6^ times bigger than the total mass of the structure [42,43,44,45,46].

Projecting the Equation (5) into the eigenspace [39], one obtains the following equation:

(6) $${\ddot{q}}_{n}+\frac{c_{n}}{m_{n}}\cdot {\dot{q}}_{n}+\frac{k_{n}}{m_{n}}\cdot q_{n}=-\frac{1}{m_{n}}\cdot \Phi_{n}^{T}\cdot P_{0}$$

where

*n*—number of modes;
m_n_, k_n_, and c_n_—vector of modal mass, modal stiffness, and modal damping for *n*-th mode;
q_n_—generalized coordinate;
ϕ_n_—*n*-th modal vector.

Adding the mass **M_0_** to the numerical model introduced rigid body modes, which are associated with extremely low frequencies (≈0 Hz) related to the movement of large mass **M_0_**. If all modes of a structure (including the rigid body ones) were considered when solving Equation (6), the total dynamic response of the structure was obtained. However, the quasi-static component of the overall structural response could be determined by only including the rigid body modes in Equation (6). The dynamic component is obtained by considering the modes associated with non-zero frequencies, i.e., omitting the rigid body modes.

### 2.4. Structural Solutions for Bridges Subjected to Dynamic Analysis

The comparative analysis was carried out for two popular types of bridges constructed in mining areas, i.e., a beam and a rigid-frame structure. Both bridges were designed based on the guidelines in the Catalog [1]. The bridges featured in the Catalog have been optimized and calculated for key (dimensioning) loads primarily derived from the traffic allowed on European roads. The authors chose bridges that were 40 m long because, according to the Catalog, they are structures most commonly encountered due to their span and load-bearing system.

The length of both bridges was 40 m, which is typical for road infrastructure in Poland. According to the Catalog, as much as 90% of road bridge structures fall within the range of maximum spans up to 40 m. Additionally, in accordance with forecasts, these types of constructions are expected to be the most frequently built in the future in Poland, considering the development of road infrastructure [47].

To limit the influence of additional parameters on the results of dynamic analyses, it was assumed that both structures have two spans, the same length (20 m) and width (13.5 m), and they are made of reinforced concrete. Additionally, both facilities were designed for the same road class (the same traffic load).

#### 2.4.1. Structural Design and Numerical Model of a Beam Bridge

**Figure 4:**
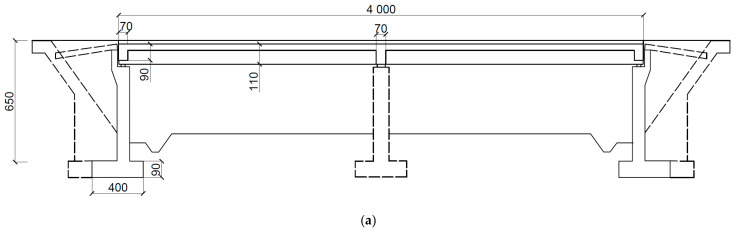

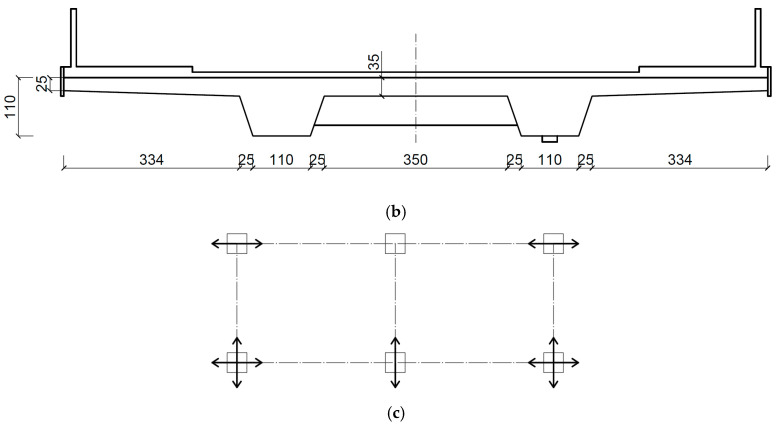
The structural details of a beam bridge (dimensions in cm): (**a**) side view; (**b**) cross-section of the slab in the midspan (left) and at the support zone (right); (**c**) the system of bearings.

The detailed geometry of a two-span beam bridge is presented in Figure 4a,b. Both bridge spans are 20 m long. The total height of the bridge is 7.4 m. Two continuous trapezoidal concrete girders integrated with the concrete slab form the bridge primary structural system. Girder beams are pre-stressed by steel tendons. The height of each girder was 1.1 m. The reinforced concrete slab was 13.5 m wide and 0.35 m thick. At each support section, the girders were stiffened with a 0.7-meter-wide cross-beam. The superstructure of bridge was supported on six bearings (Figure 4c). A set of fixed and transversally guided sliding bearings was used over the middle supports, whereas a set of longitudinally guided and multidirectional sliding bearings was applied at the side supports. The superstructure and pre-stress tendons were made of the C40/50 concrete class and Y1860 steel, respectively.

Based on the presented geometry of the beam bridge, a numerical model was created in Abaqus 2022 Software [48]. It is worth noting that certain simplifications were made in the numerical model to reduce the number of finite elements, accelerate calculations, and minimize the risk of potential numerical errors. Elements such as pillars and abutments, as well as non-structural elements including barriers and railings, were omitted. Due to the structural scheme and the way that the bridge was supported, the omission of these elements did not affect the quantitative or qualitative assessment of the dynamic response of the structure.

Structural members, such as the bridge deck, girder beams, and cross-beams, were modeled using C3D8R elements available in the finite element library of Abaqus. The applied elements were 8-node brick elements with linear shape functions and hourglass control. The total number of finite elements in the beam bridge model was approximately 40,000.

A linear-elastic material model was applied to all elements in the model. Considering the significant amount of steel in the girders, average values of the elasticity modulus for concrete and steel materials were used in the numerical model. The elasticity modulus was calculated based on the percentage of steel in the concrete and scaled up to the value of E = 41 GPa. For dynamic analyses, a Poisson ratio of v = 0.2 and a density of *ρ* = 2500 kg/m^3^ were adopted.

Boundary conditions specified at the support locations corresponded to the bearing arrangement presented in Figure 4c. To model the bearing behavior, connector elements that allow for different sliding directions were used.

**Figure 5:**
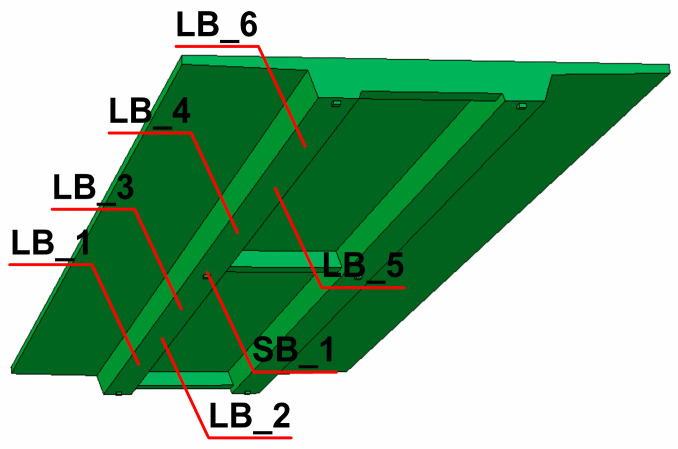
The numerical model of the beam bridge with representative elements selected for the dynamic analysis.

The completed numerical model of the beam bridge with elements chosen for detailed dynamic analyses is shown in Figure 5 (LB_1 to LB_6 for span and SB_1 for support areas, respectively).

#### 2.4.2. Structural Design and Numerical Model of a Rigid-Frame Bridge

**Figure 6:**
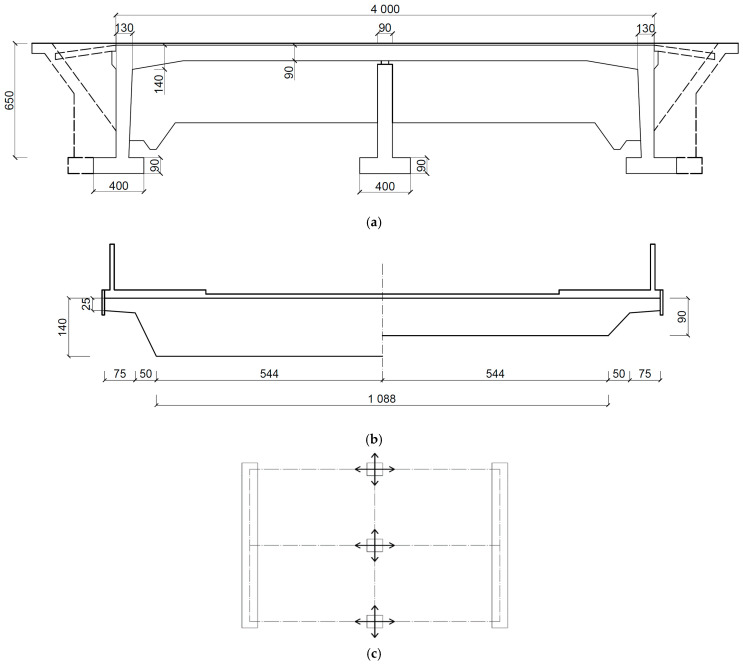
The structural details of the rigid-frame bridge (dimensions in cm): (**a**) side view; (**b**) cross-section of the slab at the support zone (left) and in the midspan (right); (**c**) the system of bearings.

The second analyzed bridge was also a two-span reinforced concrete frame structure with rigid nodes. The detailed geometry of the bridge is presented in Figure 6a,b. The length of each span was 20 m. The total height of the bridge was 7.4 m. The slab of the deck was monolithically integrated at both ends with the concrete walls. The thickness of the slab ranged from 0.9 m in the middle part of the structure to 1.4 m at the extreme supports. The widths of the deck and walls are 13.5 m. The bridge deck was additionally supported in the midspan by three round pillars with diameters of 1.2 m. Each support was placed on a foundation slab with a base dimension of 4 × 5 m and a height of 1.1 m. On the intermediate pillars, the deck was supported by a system of pot multidirectional sliding bearings (see Figure 6c). All structural elements were made of C30/37 concrete class with the following mechanical parameters: the elastic modulus E = 37 GPa, the Poisson ratio v = 0.2, and the mass density *ρ* = 2500 kg/m^3^.

**Figure 7:**
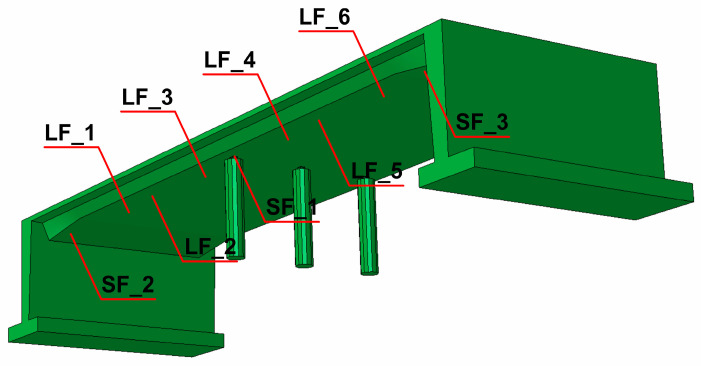
The numerical model of the rigid-frame bridge with representative elements selected for dynamic analysis.

Based on geometry and material data, the numerical model of the rigid-frame bridge was created (Figure 7) using Abaqus Software [48]. The structural elements of the bridge (deck, side walls, pillars) were modeled with approximately 120,000 finite elements. The C3D8R brick elements were used.

The finite elements of the numerical model of the rigid-frame bridge were also assigned a linear-elastic material model. The following mechanical parameters were applied for all finite elements: the elastic modulus E = 37 GPa, the Poisson ratio v = 0.2, and the mass density *ρ* = 2500 kg/m^3^.

The numerical model of the rigid-frame bridge was fully fixed at all supports. Connector elements allowing for multidirectional sliding were employed to simulate the behavior of the bearing located on the central pillars.

The numerical model of the rigid-frame bridge with elements chosen for detailed dynamic analyses is shown in Figure 7 (LF_1 to LF_6 for span and SF_1 to SF_3 for support areas, respectively).

## 3. Results

### 3.1. Numerical Evaluation of Natural Frequencies and Modes of Vibration of the Bridges

**Figure 8:**
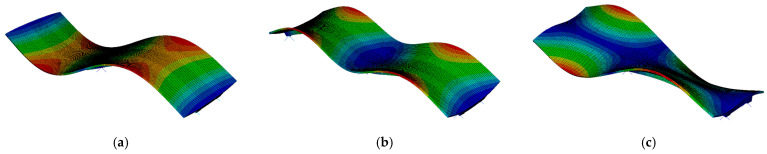
Modes of vibrations for the beam bridge: (**a**) first (vertical), (**b**) second (vertical), and (**c**) third (torsional). The color scale represents displacement from blue (zero) to red (maximum displacement).

**Figure 9:**
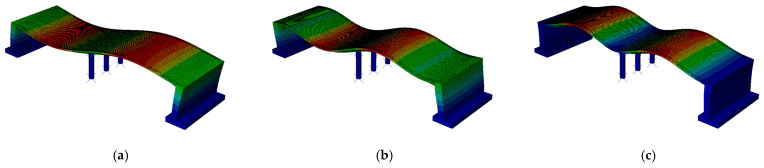
Modes of vibrations for the rigid-frame bridge: (**a**) first (longitudinal), (**b**) second (vertical), and (**c**) third (vertical). The color scale represents displacement from blue (zero) to red (maximum displacement).

The natural frequencies determined for both the beam and rigid-frame bridges are compared in Table 2, whereas modes of natural vibrations are presented in Figure 8 and Figure 9. The colors in Figure 8 and Figure 9 represent the magnitude of the modal displacement [48]. The red color represents the absolute maximum displacement (which is 1 for a normalized mode shape), whereas the blue color refers to zero displacement. Green color marks displacements in between.

The modal analysis reveals that the first eigenvalue for both bridges is very similar, measuring 4.12 Hz for the beam bridge and 4.04 Hz for the rigid-frame one. Although the first natural frequency is similar for both types of bridge, the corresponding modes of vibration differ. The first mode for the beam bridge is associated with the vertical direction, whereas for the rigid-frame bridge, the mode represents longitudinal vibration. This inconsistency results from the differences in bridge construction types. Further comparison of the modes indicates that the 2nd and 3rd modes of the rigid-frame bridge, related to vertical vibration, correspond to the 1st and 2nd modes obtained for the beam bridge.

Additionally, it also can be seen in Table 2 that the obtained natural frequencies are higher than the dominant frequency of mining shock, which is used as kinematic excitation (see Figure 1, Figure 2 and Figure 3). Thus, it can be expected that the selected bridges will operate out of the resonance zone.

### 3.2. Time History Analysis of Principal Stresses in the Bridges Subjected to the Mining Shock

The Time History Analyses of the bridges subjected to mining-induced shock (see Section 2.1) were conducted with ABAQUS software [48]. Both uniform and non-uniform models of kinematic excitation were applied in the calculations. For the non-uniform model, the wave velocities of 250, 500, and 1000 m/s were used. The maximal principal stresses in selected elements of the structures were taken as measures of dynamic response levels in the comparative analysis.

**Figure 10:**
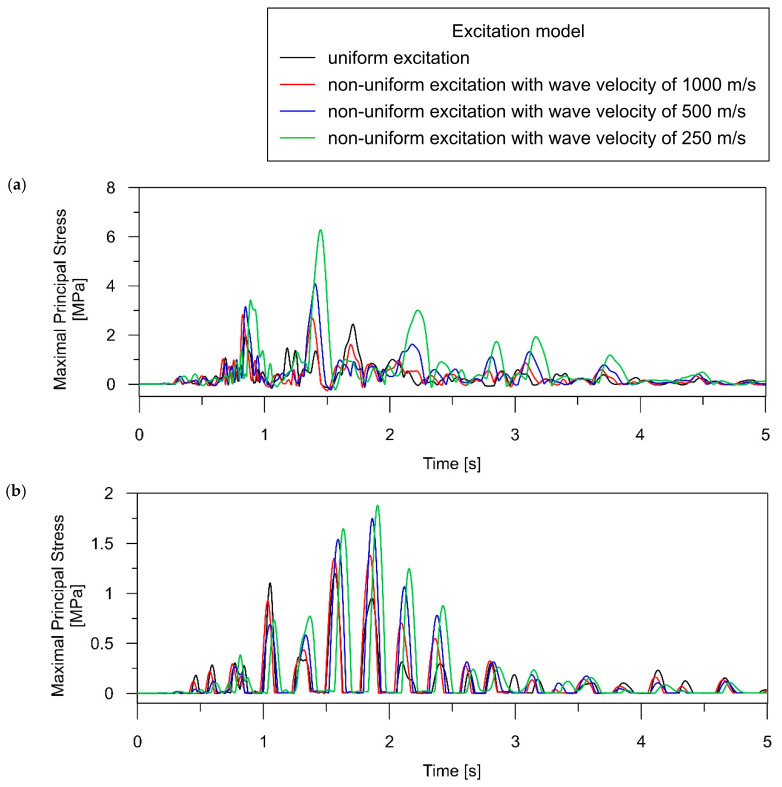
Time histories of maximal principal stress (i.e., the maximal tensile stress in the concrete material) for the beam bridge, occurring during the intense phase of seismic shock (5 s): (**a**) over the central support (element SB_1); (**b**) in the middle of the span (element LB_2).

**Figure 11:**
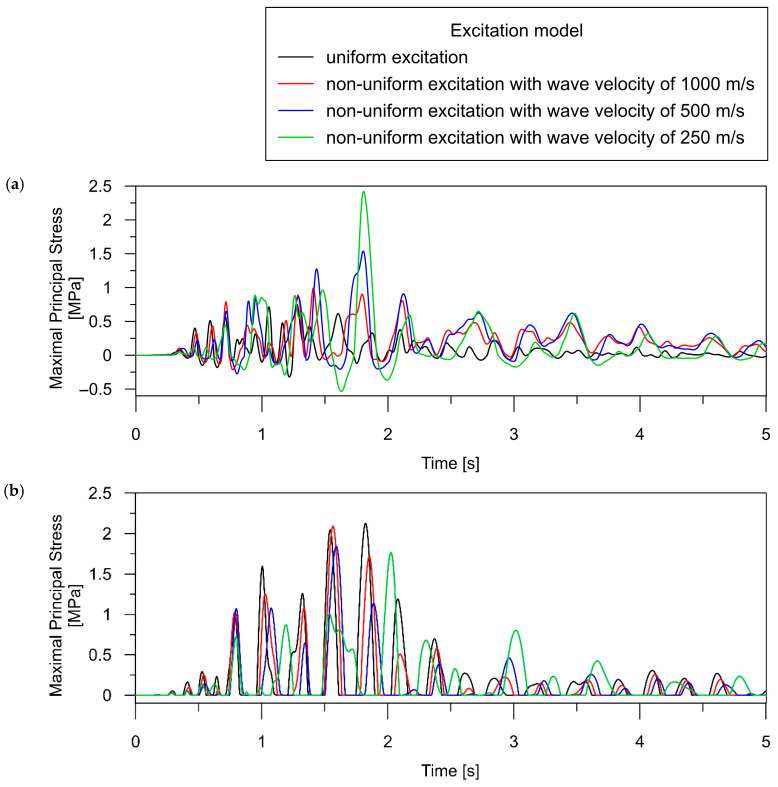
Time histories of maximal principal stress (i.e., the maximal tensile stress in the concrete material) for the rigid-frame bridge, occurring during the intense phase of seismic shock (5 s): (**a**) over the central support (element SB_1); (**b**) in the middle of the span (element LB_2).

The time histories of maximal principal stress obtained for both the uniform and non-uniform models of excitation (with various wave velocities) are presented in Figure 10 and Figure 11 for the beam and rigid-frame bridges, respectively. Based on the presented stress-time distribution, it can be concluded that the type of excitation model significantly influenced the dynamic behavior of the analyzed bridges.

In the case of the beam bridge, the following trends can be detected:

1. For point SB_1, located above the support, the maximum stress caused by the tremor is higher for the non-uniform model application (see Figure 10a) compared to the uniform model. We can observe that reducing the velocity of the shock wave results in an increase in dynamic response. It is worth emphasizing that the differences in the dynamic performance of the bridge under various excitation models are significant. The stresses obtained for two extreme excitation scenarios (uniform and non-uniform with a velocity of 250 m/s) differ 2.5-fold.
2. Applying the non-uniform excitation model results in elevated stress levels compared to the uniform excitation model, as seen in element LB_2 (refer to Figure 10b). The maximum stress for non-uniform excitation with a velocity of 250 m/s is approximately 1.5 times higher than the stresses obtained for uniform excitation.

In the case of the rigid-frame bridge, the following tendencies can be observed:

1. The stress analysis of the rigid-frame bridge in the support zone leads to both qualitative and quantitative conclusions analogous to those determined in the case of the beam bridge. For point SF_1, the maximum stress is higher with the application of the non-uniform model (see Figure 11a) compared to the uniform model, and a decrease in the wave velocity corresponds to an increase in the dynamic response. The stresses obtained for the uniform and non-uniform model with a velocity of 250 m/s exhibit 3-fold differences.
2. The non-uniform excitation model produces distinct performances in the span zones compared to the supports. The maximum stress at the midspan point LF_2 is higher for the uniform model than for the non-uniform model of excitation (see Figure 11b). The maximum stress values decrease as the wave velocity decreases, reaching the smallest value for the slowest wave at 250 m/s. The maximum stress for non-uniform excitation with a velocity of 250 m/s is approximately 20% lower than for the stresses obtained for uniform excitation.

### 3.3. Dependence of the Dynamic Behavior of the Bridges on Wave Velocity

To assess the bridges’ performances under different excitation models (i.e., uniform and non-uniform with wave velocities of 250, 500, and 1000 m/s), the total stresses in both bridges were calculated in representative elements. Then, the obtained total stresses were decomposed into the quasi-static and dynamic components. The decomposition was carried out based on the Large Mass Method algorithm (see Section 2.3).

When analyzing the diagrams presented in Section 3.3 concerning the decomposition of overall stresses into quasi-static ($\sigma_{q-s}$) and dynamic ($\sigma_{dyn}$) components, two issues must be remembered. Firstly, stresses resulting from quasi-static and dynamic effects usually reach maximum values at different times. Consequently, the total stress ($\sigma_{tot}$) does not represent the algebraic sum of its quasi-static and dynamic components. Secondly, the quasi-static and dynamic stress components may exhibit different signs at a given point in time. As a result, the total stress, expressed as the difference between these two components, may take values lower than those of the individual components. Therefore, a scenario in which the ratio $\sigma_{q-s}/\sigma_{tot}$ exceeds the value of 1.0 is justified.

#### 3.3.1. Stress–Velocity Dependence for the Beam Bridge

**Figure 12:**
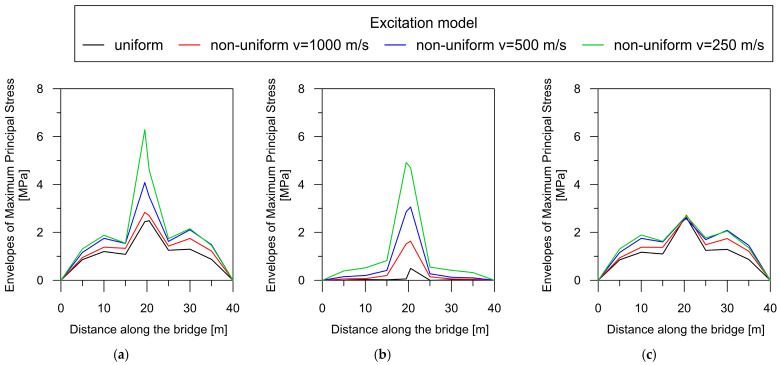
The maximum principal stress envelope plot along the beam bridge axis: (**a**) the total response; (**b**) the quasi-static component; (**c**) the dynamic component.

The dependence of the total maximum principal stress, as well as their quasi-static and dynamic components, on the wave velocity can be clearly demonstrated by creating a maximum principal stress envelope plot along the bridge axis for each model of excitation (Figure 12).

For the beam bridge, the correlation between maximal principal stresses and wave velocity is unequivocal: a reduction in the velocity of wave propagation results in a concurrent increase in the overall response of the structure (Figure 12a). Therefore, the highest stress values occurred for the lowest wave velocity of 250 m/s (green line). It can also be observed that the effect of excitation non-uniformity mostly affects the central support, where the greatest increase in the dynamic response occurred under the non-uniform model of excitation. The same dependence of both quasi-static and dynamic stress levels on the wave velocity can be observed in Figure 12b,c, respectively. The greatest increase in the quasi-static component with decreasing wave velocity occurred above the central support. However, in the case of the dynamic component, stresses were elevated based on the decrease in velocity, mainly in the span areas.

**Figure 13:**
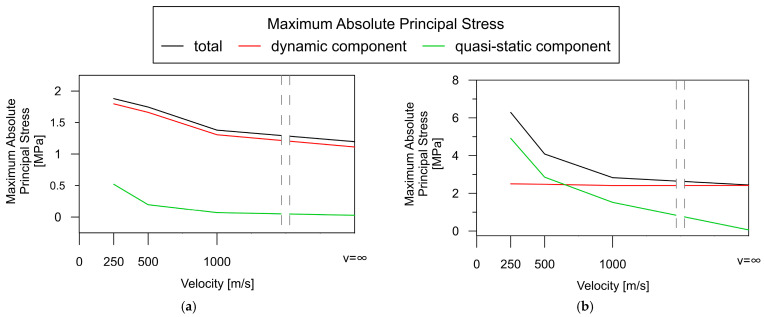
Dependence of the absolute maximum of the total principal stresses, as well as their quasi-static and dynamic components, on wave velocity for the beam bridge: (**a**) in the middle of the span (element LB_2); (**b**) over the central support (element SB_1).

To assess the contribution of quasi-static effects to the global dynamic response of the beam bridge, the quasi-static and dynamic components were compared to the total stresses shown in Figure 13 (as a function of wave velocity). The comparison was conducted for two representative elements: LB_2, situated in the middle of the span, and SB_1, located over the central support.

Based on the results in Figure 13a, the dynamic component (red line) plays a crucial role in the total stress level (black line) in the beam bridge spans. In the contrary, the quasi-static component (green line) hardly contributes to the overall response of the structure. Their values remain close to 0, except for a slight increase at the lowest wave velocity of 250 m/s.

A different trend can be observed in the support zones of the beam bridge (Figure 13b). There is an evident tendency that indicates a continuous increase in the quasi-static stress level with a decrease in wave propagation velocity (green line). Assuming that the wave velocity is less than 500 m/s, the quasi-static component constitutes much of the total response. The dynamic component of the total structural response appears to be independent of the wave velocity (red line).

#### 3.3.2. Stress–Velocity Dependence for the Rigid-Frame Bridge

**Figure 14:**
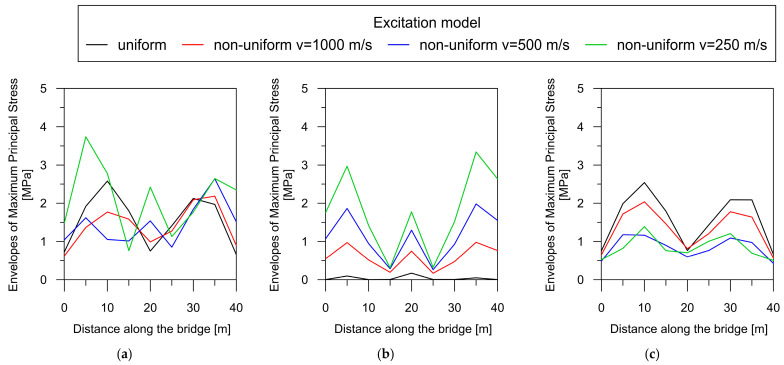
The maximum principal stress envelope for the rigid-frame bridge: (**a**) the total response; (**b**) the quasi-static component; (**c**) the dynamic component.

The dependence of the total maximum principal stress, as well as the quasi-static and dynamic components, for the rigid-frame bridge is presented in Figure 14.

For the rigid-frame bridge, the relationship between the total dynamic response and wave velocity (Figure 14a) is less clear compared to that for beam bridge (see Figure 12a). The highest total stress values occurred at the lowest wave velocity of 250 m/s (green line). However, the second-highest stress is produced under the uniform model of excitation (black line).

In turn, the correlation between the quasi-static component and the wave is more evident. There is an apparent trend indicating an increase in quasi-static stress levels with a decrease in wave propagation velocity (Figure 14b). The highest values of the quasi-static component occurred for the lowest wave velocity of 250 m/s (green line). It can also be observed that the effect of excitation non-uniformity mostly affects the support zones, where the greatest increase in the quasi-static component occurred. In the case of uniform excitation (black line), the quasi-static stresses did not appear. However, some values of the quasi-static component already occur at a high wave velocity of 1000 m/s, typical for very stiff soils.

Finally, the application of the non-uniform excitation model results in a distinct decrease in the dynamic stress component compared to the uniform model (Figure 14c). The greatest reduction is observed in the middle of the spans. The use of low wave velocities (500 m/s and lower) leads to a substantial decrease in the dynamic component of approximately 50%.

**Figure 15:**
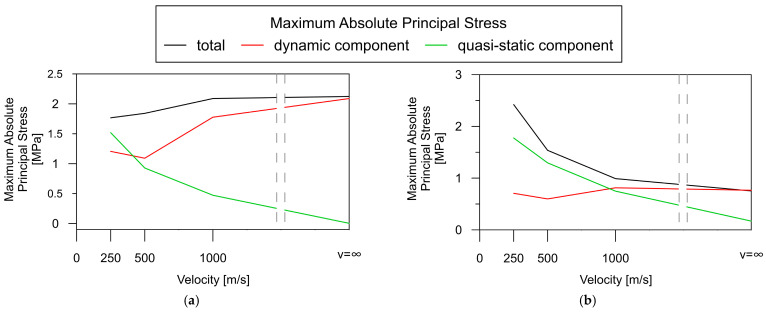
Dependence of the absolute maximum of the total principal stresses and their quasi-static and dynamic components on wave velocity for the rigid-frame bridge: (**a**) in the middle of the span (element LF_2); (**b**) over the central support (element SF_1).

The assessment of the contribution of the quasi-static effect to the overall dynamic response of the rigid-frame bridge is illustrated in Figure 15 for two representative elements: LF_2, situated in the middle of the span, and SF_1, located over the central support.

Generally, the total response (black line) in the span areas (Figure 15a) is smaller for low wave velocities (less than 1000 m/s) than for high wave velocities (over 1000 m/s). The reverse relationship occurs in the case of support zones (Figure 15b), i.e., the total dynamic response significantly increases at low wave velocities and far exceeds the response for high velocities.

Based on the results, it can be observed that the quasi-static component (green line) only plays a significant role in the total response in the span areas of the rigid-frame bridge (black line) for low wave velocities (Figure 15a). In the support zones, the contribution of the quasi-static component (green line) is dominant and close to the overall dynamic response (red line) for all wave velocities (Figure 15b). Moreover, the magnitude of the quasi-static component (green line) may exceed that of the dynamic one (red line). This trend is observed in both the span zone (for wave velocities less than 500 m/s) and the support zone (for velocities less than 1000 m/s).

#### 3.3.3. The Obtained Results in the Context of the Literature Research

To validate the obtained results, some studies (both experimental and numerical) provided by other authors are presented in detail. According to the authors’ knowledge, there are only few publications related to the impact of non-uniform excitation induced by mining activities on dynamic response, especially of rigid-frame or beam-like bridge structures. However, the results obtained in this study can be compared with the findings of other authors for different types of bridge structures.

Sextos et al. [49] analyzed the 694-meter-long Evripos Bridge in Greece, featuring a main cable-stayed span of 395 m. The bridge exhibited both the amplification and de-amplification of its response when subjected to spatially varying ground motion. The response was reduced by 10–40% under non-uniform excitation, attributed to the presence of a very thin deck, with the quasi-static component contributing less than 10%. One of the most negative effects of asynchronous excitation became of minor importance. For stiffer decks, the contribution of the quasi-static component would increase under asynchronous motion, making the effect of SVEGM more critical. However, in the case of such a flexible deck that naturally accommodates differential ground displacements more easily, the transverse displacements of the deck near the top of the pylons experienced a 31% amplification under non-uniform excitation.

Then, Harichandran [50] conducted analyses of three structurally different bridges, which included a suspension bridge and two arch bridges. For the suspension bridge, uniform excitation significantly over-estimated responses at the longer main and under-estimated responses at other locations. In turn, for the arch bridges, uniform excitation under-estimated forces in the arch and over-estimated forces in the deck.

An analysis of a 300-meter-long six-span continuous deck reinforced concrete bridge was conducted by Monti et al. [24]. The authors observed that the overall effect of spatial variability was to reduce the dynamic response at the central piers and increase it at the lateral ones.

The analysis of a 184-meter-long five-span bridge was presented by Tzanetos et al. [23]. Depending on the characteristics of spatially varying excitation, the bridge configuration, and its boundary conditions, asynchronous ground motion can induce a higher response in the structure than the response resulting from the uniform motions of its supports.

Zerva [8] conducted an extensive review of studies related to the dynamic analysis of bridges concerning the influence of non-uniform excitation on their dynamic response. The most significant conclusion from this comprehensive study is that spatially varying seismic excitation may cause a distinct response pattern in bridges compared to uniform ground motions. The quasi-static effects contribute to additional deformation in structures, potentially increasing the overall dynamic response.

Drygala et al. [51] performed the dynamic analysis of a 4-meter-long rigid-frame footbridge under seismic excitation. The authors performed an in situ experiment to obtain the footbridge’s modal characteristics and conducted an experimental check-up of its responses to non-uniform excitation. It was revealed that non-uniform seismic excitation could result in an increase in dynamic response compared to the response observed under uniform excitation. This effect was particularly manifested near the rigid nodes of the structure, where quasi-static effects played a crucial role. The differences between results were in the range of 10 to 50%.

Finally, Boron et al. [52,53] analyzed the response of a 160-meter-long five-span road viaduct to non-uniform mining-induced excitation. The structure was examined under mining shocks recorded in two main mining activity areas in Poland. The authors validated the numerical model and determined the wave propagation velocity through an in situ experiment. The analysis demonstrated a significantly greater dynamic response in the support zones under non-uniform excitation compared to the uniform one. Conversely, the dynamic response in the spans showed higher values in the case of the uniform excitation.

In the context of the provided literature review, including both experimental and numerical studies, the results presented in this work appear to be validated and further supported.

## 4. Discussion on the Susceptibility of the Bridges to Quasi-Static and Dynamic Effects

The analysis in Section 3 allows for assessing the influence of non-uniform kinematic excitation resulting from mining-induced shock on the dynamic behavior of two distinctly designed bridges. The contributions of quasi-static and dynamic stresses to the overall dynamic responses are compared, and the structures’ susceptibility to changes in ground wave velocity are observed. Furthermore, this study identifies the zones of the structures that are most vulnerable to quasi-static effects.

### 4.1. Rigid-Frame Bridge vs. Beam Bridge: A Comparison of Susceptibility to Quasi-Static Effect

**Figure 16:**
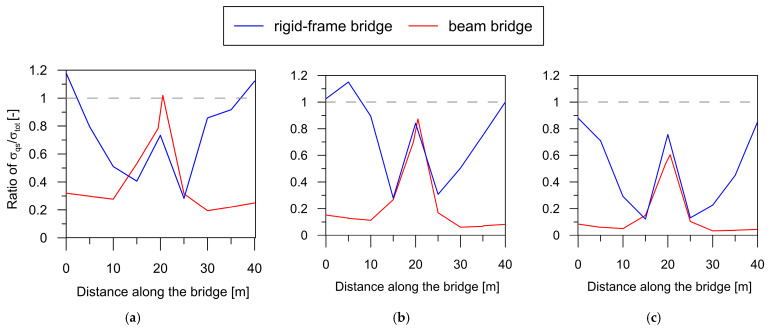
Envelope of ratio of $\sigma_{q-s}/\sigma_{tot}$ for both bridge types for non-uniform excitation with velocities of (**a**) 250 m/s, (**b**) 500 m/s, and (**c**) 1000 m/s.

To compare the impact of the quasi-static component on the overall structural response for both bridge types, the ratios of maximum quasi-static stress to maximum total stress ($\sigma_{q-s}/\sigma_{tot}$) are shown in Figure 16 for wave velocities of 250, 500, and 1000 m/s.

An important observation derived from the comparative analysis in Figure 16 is that the quasi-static component significantly contributes to the total dynamic responses of both the rigid-frame bridge (blue line) and the beam bridge (red line). The $\sigma_{q-s}/\sigma_{tot}$ ratios, taking values over 50% in the support zones of the structures, highlight the substantial role played by the quasi-static component in the overall dynamic responses.

However, it is evident that the rigid-frame structure is more profoundly affected by quasi-static effects. In this structure, the highest contribution of quasi-static stress was observed around the extreme supports of the structure, where the ratio $\sigma_{q-s}/\sigma_{tot}$ was about 1.0. In the span zones, the ratio was up to 0.4 in the case of low wave velocities. Comparatively, in the beam bridge, the zone of increased quasi-static stresses was confined to the central support (with the $\sigma_{q-s}/\sigma_{tot}$ ratio ranging from 0.5 for stiff soils to 1 for soft soils), leaving the outer zones practically unaffected by quasi-static effects. The reason for this lies in the difference in stiffness between these two types of bridges. A beam bridge presents a considerably less stiff structure than a rigid-frame one, especially in the vicinity of the outer supports; thus, lower quasi-static effects were observed.

In general, it can be concluded that rigid-frame bridges with high stiffness exhibited much greater susceptibility to quasi-static effects resulting from the non-uniform excitation of mining origin compared to beam bridges.

### 4.2. Rigid-Frame Bridge vs. Beam Bridge: A Comparison of Susceptibility to Dynamic Effects

**Figure 17:**
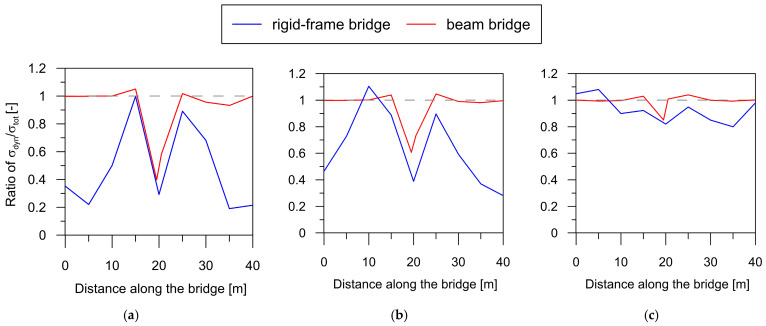
Envelope of ratio of $\sigma_{dyn}/\sigma_{tot}$ for both bridge types for non-uniform excitation with velocities of (**a**) 250 m/s, (**b**) 500 m/s, and (**c**) 1000 m/s.

To observe the influence of the dynamic component on the total response for both bridge types, the ratios of maximum dynamic stress to maximum total stress ($\sigma_{dyn}/\sigma_{tot}$) are presented in Figure 17 for wave velocities of 250, 500, and 1000 m/s.

Based on the comparative analysis in Figure 17, it is evident that the dynamic component plays a more substantial role in the overall response of the beam bridge (red line) compared to the rigid-frame bridge (blue line).

Additionally, the contribution of the dynamic component to the total stress ratio is strongly influenced by the wave velocity. For the rigid-frame bridge situated on softer soils (with wave velocities of 250 and 500 m/s), the dynamic component in support areas contributes less than 40% to the overall structural response. However, in spans, this contribution reaches 100%. In the case of the beam bridge, the dynamic component accounts for 100% along the entire structure, except the central support.

On the other hand, for stiffer soils (with a wave velocity of 1000 m/s), the dynamic component covers 80–100% of the total response for both bridge types.

The observations above show that the beam bridge is more prone to the dynamic component than the rigid-frame bridge.

## 5. Conclusions

In this paper, the dynamic analysis of two bridges (rigid-frame bridge and beam bridge) exposed to spatially varying mining-triggered tremors was presented. The influence of wave propagation velocity in the ground on the dynamic responses of the structures was also examined. Finally, the susceptibility of both bridges to the quasi-static and dynamic stress components was assessed and compared. Based on the conducted research the following conclusions can be drawn:

- The dynamic responses of both types of bridges undergo significant changes when subjected to the model of spatially varying ground mining-induced excitation, compared to the model of uniform excitation.
- In the case of the beam bridge, the impact of non-uniform excitation is evident across the entire structure, with the most notable effects observed at the central support, where the dynamic response evidently increases with decreasing wave velocity.
- The relationship between the total dynamic response and the wave velocity is less straightforward for the rigid-frame bridge. Similarly, as observed in the case of the beam bridge, applying the spatially varying excitation model results in increased stress in the support zones. However, in the spans, the highest stresses are obtained under uniform excitation.
- The quasi-static component plays a crucial role in the overall dynamic responses for both beam and rigid-frame bridges. However, rigid-frame bridges exhibit much greater susceptibility to quasi-static effects compared to beam bridges. This is related to the higher stiffness of the rigid-frame bridge. On the other hand, beam bridges are more susceptible to the dynamic components of stresses.

In general, the presented conclusions can be helpful for choosing the type of bridge construction with lower susceptibility to the non-uniformity of excitations for areas affected by mining tremors.

In the case of spatially varying ground excitation, both quasi-static and dynamic components always appear in the total dynamic response of a bridge of any construction. An appropriate choice should be based on the possibility of limiting the individual components of stress. In practice, reducing the quasi-static component requires strengthening the structure, especially its nodes, which is neither easy nor a cost-effective procedure. In turn, a reduction in the dynamic component can be achieved by using vibration absorbers or dampers. Fitting a structure with such equipment seems to be a more economical and less complicated process.

This study indicates that the contribution of the quasi-static and dynamic components in the global response depends on the geological and soil conditions on which the bridge is founded. If a bridge is built on weak ground (wave velocity 250–500 m/s), significant quasi-static effects can be expected, especially in the support zones. At the same time, dynamic effects are reduced. This applies to both types of the analyzed bridges. However, the conducted studies make it clear that in a bridge with greater stiffness, larger quasi-static effects will occur. Hence, it is advisable to avoid stiffer rigid-frame structures in such weak ground. The necessity of strengthening them may result in higher construction costs. Furthermore, the dynamic component of stress constitutes only a minor fraction of the overall structural responses in rigid-frame structures, making the use of vibration dampers largely ineffective. In that situation, constructing a beam bridge is a better recommendation, as the reduction in stress levels (if needed) in the structure due to the use of damping devices will be much more evident. The dynamic component of stress for beam bridges is a larger part of the total structural response, and the reduction in this component leads to a substantial decrease in overall stresses.

The presented work provides some insight into the susceptibility and structural behaviors of typical beam and rigid-frame bridges under mining-induced seismic excitations, making it potentially applicable in engineering practice. In the context of further research, the authors plan to experimentally validate the obtained results through in situ investigations and shaking table tests, taking into consideration bridges constructed according to the specifications outlined in the catalog of typical constructions of road bridge structures.

## Figures and Tables

**Table 1:** Comparison between natural and mining-induced seismic shocks [30,31,32,33].

Basic Characteristics	Natural Seismic Shocks	Minig-Induced Seismic Shocks
Source of the shock	Natural tectonic processes	Human activities related to mining, such as the extraction of minerals
Intensity and occurrence frequency	High magnitudes, frequency varying due to geological factors	Lower magnitudes but frequent in areas with extensive mining operations
Influence range	Widespread; significant environmental impacts, including infrastructure damage and life loss	Up to 10 km; primarily affect the mining area, potentially causing ground instability and damage to mine infrastructure
Shock duration	Lasts in minutes	Lasts up to 15 s
Intense phase time	Minutes	0.5–5 s
Dominant frequency	Low range: 0.5–2 Hz	Higher range: 2–7 Hz
Seismic wave arrival sequence	Different types of seismic waves (primary, shear, and Raleigh) reach the receiver sequentially	Due to the proximity of the source, all types of body waves, along with surface waves, arrive at the receiver almost simultaneously
Magnitude of shock spatial components	The greatest amplitudes occur in the horizontal direction, parallel to the Raileigh wave propagation	Amplitudes in three directions are comparable; vertical amplitudes may even exceed those of horizontal vibrations
Decay rate of the shock	Decrease in vibration amplitudes depends on the geological site conditions	Impulse-like nature of amplitudes; the decay in amplitudes with increasing distance from the source occurs much more rapidly
Acceleration and frequency content	Acceleration and frequency range predictable, typical for given energies and epicentral distances	Unpredictable, wide acceleration and frequency range for given energies and epicentral distances

**Table 2:** Comparison between the natural frequencies of the rigid-frame and beam bridges.

Mode	Natural Frequency [Hz]	
	Beam Bridge	Rigid-Frame Bridge
I	4.12	4.04
II	6.18	7.97
III	6.97	8.48
IV	7.38	10.96

## Data Availability

Data are contained within the article.
